# Epigenetic Silencing of *PTEN* and Epi-Transcriptional Silencing of *MDM2* Underlied Progression to Secondary Acute Myeloid Leukemia in Myelodysplastic Syndrome Treated with Hypomethylating Agents

**DOI:** 10.3390/ijms23105670

**Published:** 2022-05-18

**Authors:** Paul Lee, Rita Yim, Kai-Kei Miu, Sin-Hang Fung, Jason Jinyue Liao, Zhangting Wang, Jun Li, Yammy Yung, Hiu-Tung Chu, Pui-Kwan Yip, Emily Lee, Eric Tse, Yok-Lam Kwong, Harinder Gill

**Affiliations:** 1 Department of Medicine, School of Clinical Medicine, LKS Faculty of Medicine, The University of Hong Kong, Hong Kong, China; pl85@hku.hk (P.L.); ritayim@hku.hk (R.Y.); u3558354@hku.hk (Y.Y.); u3557654@hku.hk (H.-T.C.); u3557642@hku.hk (P.-K.Y.); emilylmy@connect.hku.hk (E.L.); ewctse@hku.hk (E.T.); ylkwong@hku.hk (Y.-L.K.); 2 School of Biomedical Sciences, Faculty of Medicine, The Chinese University of Hong Kong, Hong Kong, China; kelvinmiu@cuhk.edu.hk (K.-K.M.); shfung@link.cuhk.edu.hk (S.-H.F.); wangzhangting@cuhk.edu.hk (Z.W.); 3 Department of Chemical Pathology, Faculty of Medicine, The Chinese University of Hong Kong, Hong Kong, China; liaojinyue@cuhk.edu.hk; 4 Department of Infectious Diseases and Public Health, The City University of Hong Kong, Hong Kong, China; jun.li@cityu.edu.hk

**Keywords:** PTEN, MDM2, myelodysplastic syndrome, hypomethylating agents, resistance

## Abstract

In myelodysplastic syndrome (MDS), resistance to hypomethylating agents (HMA) portends a poor prognosis, underscoring the importance of understanding the molecular mechanisms leading to HMA-resistance. In this study, P39 and Kasumi-1 cells and their azacitidine-resistant and decitabine-resistant sublines were evaluated comparatively with transcriptomic and methylomic analyses. Expression profiling and genome-wide methylation microarray showed downregulation of *PTEN* associated with DNA hypermethylation in P39 cell lines resistant to azacitidine and decitabine. This pattern of *PTEN* dysregulation was also confirmed in a cohort of patients failing treatment with HMA. DNA hypomethylation of *MDM2* was detected with downregulation of *MDM2* in HMA resistant cell lines. Long-read sequencing revealed significant RNA hypomethylation of *MDM2* resulting in alternative splicing and production of a truncated *MDM2* transcript in azacitidine-resistant P39 cells. The expression of this *MDM2* truncated transcript was also significantly increased in HMA-resistant patients compared with HMA-responsive patients. In conclusion, epigenetic and epi-transcriptomic dysregulation of *PTEN* and *MDM2* were associated with resistance to hypomethylating agents.

## 1. Introduction

Myelodysplastic syndrome (MDS) is a clonal myeloid neoplasm characterized by multiple genetic aberrations and complex clonal architectures [1]. These diverse genetic aberrations account for the different clinical presentations, including treatment responses, survivals, and risks of progression to acute myeloid leukemia (AML) [2]. The hypomethylating agents (HMA) azacitidine (AZA) and decitabine (DEC) are standard therapeutic options in patients with higher-risk MDS [3]. Therapeutic objectives in MDS include ameliorating cytopenia and related complications, reducing transfusion requirement, enhancing quality of life, lowering risks of secondary AML, and improving survivals [4,5].

Responses to AZA and DEC in patients with MDS and AML with myelodysplasia-related changes are generally short-lived [6]. Somatic mutations of *ASXL1*, *TET2,* and/or *DNMT3A* are associated with a hypermethylated genomic profile, which alters responses to HMA [6,7,8,9]. Studies at the messenger RNA (mRNA) level showed that resistance to AZA was associated with downregulation of uridine-cytidine kinase (*UCK*), an enzyme required for AZA activation upon cellular uptake [10]. In studies evaluating resistance to DEC, a higher expression ratio of cytidine deaminase (*CDA*) to deoxycytidine kinase (*DCK*) impaired mono-phosphorylation and hence activation of DEC, with accelerated deamination of DEC leading to faster rate of DEC inactivation [11]. Targeted methylation analyses also showed hypomethylation of *LINE1*, *PGRB,* and *miR-124a-3*, but the pathogenetic roles of these genes remain unclear [11].

Although gene dysregulations associated with HMA resistance have been widely studied, a comprehensive approach that correlates somatic mutations and epigenetic alterations with gene activities may reveal important mechanisms accounting for HMA resistance [6,12,13]. In this study, we employed a multi-omics approach to identify biologic pathways altered during HMA resistance in cell lines and patient samples. In addition to genome-wide methylation analysis, the transcriptome and epitranscriptome were evaluated for expression, somatic mutations, and RNA methylation, aiming to correlate transcriptomic dysregulation with genetic, epigenetic, and epi-transcriptional signatures.

## 2. Method

### 2.1. Cell Lines and Cell Cultures

P39/Tsugane (courtesy of Prof Yoshida Takeda, Japan) and Kasumi-1 (DSMZ) cell lines were cultured using medium RPMI-1640 glutamax-1 with 10% fetal bovine serum (FBS) and RPMI-1640 with 20% FBS, respectively. Penicillin (25 IU/mL) and streptomycin (25 µg/mL) were supplemented, and cells were incubated at 37 °C in humidified 5% CO_2_. To establish derivative sublines resistant to AZA and DEC, both cell lines were continuously exposed to increasing concentrations of AZA and DEC from 5nM onwards. Sublines that were resistant to ≥ 10µM of AZA or DEC were established as P39-AZA-R and P39-DEC-R, and Kasumi-1-AZA-R and Kasumi-1-DEC-R. 

### 2.2. Cell Viability Assays

To determine the IC50 of AZA and DEC for the parental cell lines and sublines, cells were seeded at a density of 1000/µL and treated with increasing doses of AZA and DEC. Cell viability was determined with presto blue stain at 24, 48, and 72 h during treatment with 2.5 nM to 2.5 mM of AZA or 1 nM to 0.1 mM DEC. The IC50s of AZA and DEC were determined. 

### 2.3. DNA Whole Methylome Analysis

The Illumina^®^ Infinium MethylationEPIC BeadChip (850 k) (Illumina, San Diego, CA, USA) was used to quantify 866,836 CpG sites in the genome according to the manufacturer’s protocol. Briefly, bisulfite conversion of genomic DNA was performed using Zymo EZ DNA methylation kit (Zymo Research, Irvine, CA, USA) and denatured for isothermal whole genome amplification. Amplified DNA was fragmented, and hybridized overnight onto BeadChip followed by washing, staining, and extension. The final chip imaging was performed on Illumina ^®^ iScan System (Illumina, San Diego, CA, USA). Bioinformatics analysis of Methylation EPIC data was performed using the Illumina ^®^ GenomeStudio^TM^ Methylation module v2011 (Illumina, San Diego, CA, USA). CpG sites passing quality controls were filtered and normalized for differential methylation calculation. Comparison between P39 (control group) and P39-AZA-R and P39-DEC-R (resistant group) lines were made with threshold above 2.5-fold or below 0.4-fold. Unsupervised clustering, Gene ontology (GO), and Kyoto Encyclopedia of Genes and Genomes (KEGG) analyses using the selected gene list with differential methylation regions (DMRs) were performed and visualized [14,15].

### 2.4. Whole Transcriptome Analysis

High quality RNA extracted from P39, P39-AZA-R, P39-DEC-R lines was enriched for mRNA by Oligo (dT) followed by preparation into whole transcriptomic library. Paired-end sequencing was performed on the Beijing Genome Institute (BGI, Shenzhen, China) next-generation sequencing platform (BGISEQ-500) to acquire at least 5 million reads at 100 bp per read. Cleaned reads pre-processed by SOAPnuke toolkit were mapped to the reference genome hg19, while spliced-aligned reads mapped by HISAT2 were subjected to StringTie and Cuffcompare to reconstruct transcripts for novel transcript prediction [16,17,18,19]. The combined transcriptomic assembly was used as reference for reads re-alignment by Bowtie2 [20] followed by gene expression level calculation with RNA-seq by Expectation Maximization (RSEM) [21].

Subsequent hierarchical clustering and Pearson correlation were analyzed using default packages cor and hclust in R and heatmaps were visualized using ggplot in R [22]. Differentially expression genes (DEG) with more than 2-fold difference between P39 and P39-AZA-R/P39-DEC-R lines were identified using the PossionDis software. Functional annotations and predictions were conducted using Gene Ontology (GO) analysis and KEGG pathway analysis to detect enriched functional GO terms, biological processes, and signaling pathways. Distribution analysis was performed using phyper to monitor threshold false discover rates (FDR), with significant enriched GO terms defined as no larger than 0.01 [15,23,24]. SNPs and INDELs were detected following the GATK method, and gene fusion events were detected using SOAPfuse [25]. High confidence variants were filtered by default parameter, and the Circos software was used to visualize gene fusions [26].

### 2.5. RNA Modifications and Alternative Splicing Detection

Native RNA libraries were prepared from P39 and P39-AZA-R lines with the direct RNA sequencing kit (Oxford Nanopore Technologies, Oxford, UK) according to the manufacturer’s protocol (SQK-RNA002). Briefly, reverse-transcribed RNA was purified and loaded into flow cell (FLO-MIN106D) for sequencing. Raw electrical signal data (.fast5) were used for base calling using Guppy (v4.0.5, Oxford Nanopore Technologies) with the high accuracy model. The quality-filtered direct RNA-seq (DRS) reads were mapped to GrCh38 reference human transcriptome with minimap2 (v2.17) utilizing default parameters that detected spliced long reads [27]. The resulting data were resquiggled with nanopolish eventalign (v0.13.2) [28]. For the detection of differential RNA modifications, xpore (v0.5.3) was adopted with default parameters [29].

### 2.6. Pyrosequencing and Quantitative Polymerase Chain Reaction

Hypermethylated or hypomethylated loci of interest were validated by pyrosequencing (Supplementary File S1). Completely methylated and completely unmethylated controls (Qiagen, MD, USA) were used and mixed in 1:1 ratio to produce a 50% methylated control. Gene expression quantification by Real Time-PCR was performed using the SuperScript IV VILO Master Mix (Thermo Fisher, Waltham, MA, USA) and PowerUp™ SYBR™ Green Master Mix (Thermo Fisher Scientific, Waltham, MA, USA). 

### 2.7. Patients and Samples

Bone marrow (BM) aspirates of 13 patients with MDS resistant to HMA and 14 MDS patients responsive to HMA were used. Resistance to HMA was defined as disease progression from MDS to secondary AML during HMA treatment, and BM samples at progression were collected. Responsiveness to HMA was defined as marrow and/or hematological improvement. BM samples at the time of progression to AML and at best response to HMA were collected. Baseline clinicopathologic and molecular characteristics of all patients were determined and reviewed before being used to validate cell line data generated in vitro. This study was approved by the Institutional Review Board of the University of Hong Kong/Hong Kong West Cluster (UW 19-634), and written informed consents were obtained. 

### 2.8. Statistical Analyses and Data Sharing

Unless otherwise specified, all grouped data were presented as mean ± standard error (SE). Statistical analyses were performed using the unpaired *t*-test or Chi-square test. Correlation between variables were determined using the Pearson correlation coefficient. Two-tailed *p*-values of <0.05 were considered statistically significant. All high-throughput sequencing data that supported the findings of this study were deposited in the Gene Expression Omnibus (GEO) with the accession code (GSE165188). For specific dataset, the accession numbers are GSE165185 for methylation microarray data, GSE165187 for RNA transcriptomic data, and GSE165064 for native RNA sequencing data for epi-transcriptomic analysis. Other relevant data supporting the key findings of this study are available within the article or upon request to the corresponding author.

## 3. Results

### 3.1. Establishment of AZA-R and DEC-R Sublines

By continuously exposing P39 and Kasumi-1 cells to AZA or DEC, P39-AZA-R and Kasumi-1-AZA-R and P39-DEC-R and Kasumi-1-DEC-R sublines were established. The IC50s of the P39-AZA-R/P39-DEC-R and Kasumi-1-AZA-R/Kasumi-1-DEC-R sublines were ≥1.5-log and ≥2 log-fold higher than those of P39 and Kasumi-1 (Supplementary Table S2). Cells resistant to AZA and DEC were harvested for whole methylome and transcriptome analysis.

### 3.2. Methylome Dysregulation during HMA Resistance in Cell Lines

**Figure 1:**
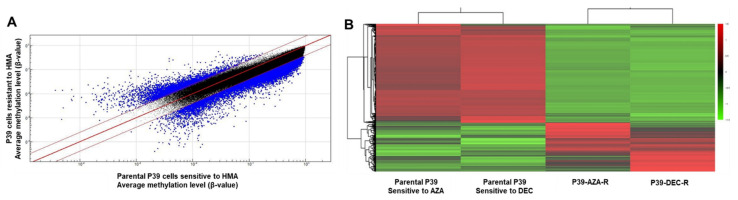
Differential methylation signature between hypomethylating agent (HMA)-resistant and sensitive P39 cells. (**A**) Scatter plot of differential methylation loci of hypomethylating agent-resistant and sensitive P39 cell lines. Average methylation levels of P39 cell line resistant to azacitidine and decitabine were compared with the mean methylation levels of P39 cells sensitive to azacitidine and decitabine. Differential methylation levels of at least 2.5-fold or 0.4-fold were included for downstream study as differentially methylated regions (DMRs); (**B**) Differential methylation signature and functional prediction of differentially methylated regions (DMRs) in HMA-sensitive and HMA-resistant cells. Hierarchical clustering of DMRs showed a global hypomethylation signature during resistance to both azacitidine (AZA) and decitabine (DEC). Methylation in AZA-resistant P39 cell line (P39-AZA-R) and DEC-resistant P39 cell line (P39-DEC-R) differed by a small number of hypermethylated genes. The sensitive parental P39 cells were treated separately with AZA and DEC at 1 µM for 48 h followed by immediate harvest for the assessment of methylation signature.

Of the 866,836 CpG sites tested, 3604 loci were hypermethylated in P39-AZA-R and/or P39-DEC-R cells, with average methylation levels of ≥2.5-fold higher than those of P39 cells. In contrast, there was at least 10-fold more hypomethylated loci (45,932 CpG sites) with average methylation level ≤ 0.4-fold in HMA-resistant cells compared with HMA-sensitive parental cells (Figure 1A). Amongst the 20,126 annotatable differential methylation regions (DMRs), 33% (i.e., 6689 loci) were classified as CpG islands while the remaining were CpG-flanking sites of either CpG shelf or shore (Supplementary Tables S3 and S4). When studying the DMRs in the context of localization to known UCSC RefGene, the majority of CpGs were embedded within gene bodies while there were 32% either in close vicinity to the transcriptional start site (TSS) or spanning the first exon (Supplementary Tables S3 and S4).

Genome-wide hypomethylation in both P39-AZA-R and P39-DEC-R was also observed in the clustering analysis with minor differences between P39-AZA-R and P39-DEC-R (Figure 1B). In particular, among the 45,932 hypomethylated CpGs, there were 42% (19,498 hypomethylated CpGs) predicted either in close vicinity to TSS or spanning the first exon of the known genes or non-coding RNAs. Excluding the 555 CpGs mapping to unclassifiable open reading frames, there were 18,941 hypomethylated CpGs mapping to known gene or non-coding RNA (Supplementary Table S5). Despite global hypomethylation signatures in both P39-AZA-R and P39-DEC-R, 88% of the hypermethylated CpGs (3170 loci) were predicted to be either in close proximity to TSS or spanning the first exon of UCSC RefGene known genes or non-coding RNA.

Among the 11,564 genes or non-coding RNA with differential methylation signatures, PTPRN2, AKNRD11, MAD1L1, RPTOR, and TNXB were the genes with the highest frequencies of differential methylation, while PTEN and MDM2 also showed significant differential methylation (Supplementary Table S6). Results from the GO and KEGG prediction analyses indicated that TP53 dysregulation was implicated. TP53 function was significantly associated with the PI3K/Akt/mTOR signaling pathway, with PTEN and MDM2 predicted to play major roles (Supplementary Table S7). In addition to PTEN and MDM2, other genes predicted to be associated with HMA resistance by GO and KEGG analyses included EGFR, p16INK4a (CDKN2A), and p14ARF, all of which were directly or indirectly regulated by PTEN and MDM2. 

### 3.3. Transcriptomic Dysregulation and Corresponding DNA Methylomic Changes in HMA-Resistant Cell Lines

**Figure 2:**
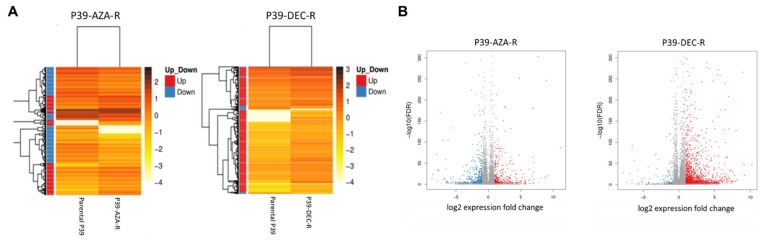
Differential expression profile of hypomethylating agent (HMA)-resistant P39 cells. (**A**) RNA expression profiles of AZA-resistant P39 (P39-AZA-R) and DEC-resistant P39 (P39-DEC-R) were distinct from each other with different clustering pattern; (**B**) Volcano plot of differentially expressed genes. Overexpression of genes was seen in P39-DEC-R and P39-AZA-R when compared with P39. The upregulated genes (blue) correspond to their negative log10 false discovery ratio (FDR) (*p*-value). P39-DEC-R (right) detected 1296 significantly upregulated genes (red dots) while P39-AZA-R (left) detected 306 significantly upregulated genes when compared with P39. There were 420 downregulated genes detected in P39-AZA-R and 89 downregulated genes detected in P39-DEC-R in comparison with P39.

**Figure 3:**
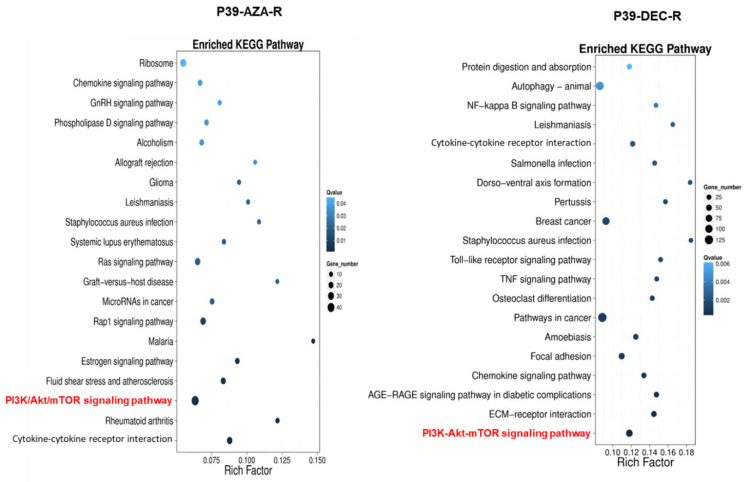
KEGG prediction for pathway and functional roles of differentially expressed genes (DEGs). Functional prediction by KEGG analysis of differentially expressed genes in hypomethylating agent (HMA)-resistant P39 sublines compared with P39 was performed separately for azacitidine-resistant P39 (P39-AZA-R) and decitabine-resistant P39 (P39-DEC-R). This gene set analysis studied both over-expressed and under-expressed genes together to look for biological pathways that are implicated. Both HMA-resistant sublines had dysregulated PI3K/Akt signaling that were predicted to be mediated by platelet-derived growth factor subunit A (PDGFA) and Endothelia PAS domain-containing protein 1 (EPAS1) upon correlation with DNA methylation patterns. Both sublines also showed similar dysregulation in cellular processes. The PI3K-Akt signaling pathway (red) was functionally significant in both cell lines. The results from this RNA expression-based analysis support the 850 K methylome data that predicted PI3K (PTEN and MDM2) signaling dysregulation during HMA resistance. Rich factor: ratio of the DEG number and the number of genes annotated in the relevant pathway. The greater of the Rich factor, the greater the degree of significance of the pathway identified. KEGG: Kyoto Encyclopedia of Genes and Genomes.

P39-AZA-R and P39-DEC-R displayed distinct transcriptomic profiles in the two clustering analyses (Figure 2A,B). There were significantly more upregulated genes in P39-DEC-R when compared with P39-AZA-R (1296 versus 306 upregulated genes), while there were only 89 downregulated genes in P39-DEC-R, 331 genes fewer than that observed in P39-AZA-R (Supplementary Tables S8 and S9). Among the downregulated genes in P39-AZA-R and/or P39-DEC-R, 10 genes were found to be hypermethylated based on whole methylome analysis, including KIFC1, CTTN, FAM171A2, EPAS1, KANK2, PDGFA, KIFC3, CDKN1C, TP53INP2, and FTH1. During functional prediction of these commonly dysregulated genes, the KEGG pathway analysis predicted pathogenic activation of multiple signaling pathways, most notably the PI3K/Akt/mTOR signaling pathway (Figure 3). GO terms classification analysis indicated that the majority of identified genes were involved in cellular processing, and approximately 50% of dysregulated genes were involved in signaling transduction via protein-protein binding or protein-nucleic acid binding activities (Supplementary Table S10).

### 3.4. Aberrant DNA Methylation Associated with PTEN and MDM2 Dysregulation in HMA-Resistant Cell Lines

Amongst all genes, dysregulation of PTEN and MDM2 and the PI3K/Akt/mTOR signaling pathway was consistently predicted to be significant by both methylomic and transcriptomic analyses. PTEN and MDM2 showed differential methylation and RNA expression in P39 during resistance to HMA and were functionally significant based on predictions made by KEGG and GSEA analyses (Figure 3 and Supplementary Table S7). PTEN and MDM2 were therefore selected for validation by pyrosequencing and real-time PCR in parental and HMA-resistant lines. Among the hypermethylated loci, cg03891929 and cg10041390 located inside the two CpG islands were approximately 200bp upstream to PTEN, and they were hypermethylated by 2.9-fold and 4.4-fold, respectively, in HMA-resistant lines. For pyrosequencing spanning cg10041390 and the eight flanking CpGs, a low level of methylation was detected in HMA-resistant P39 cells. The average methylation across all CpG loci of both P39-AZA-R and P39-DEC-R were higher than that of P39 (*p* = 0.0028 and *p* = 0.02, respectively). In particular, P39-AZA-R showed a 10–40% increase of methylation in comparison with P39, and hypermethylation of P39-DEC-R was observed across all sites analyzed except cg10041390, ranging from 3–21% (Table 1 and Supplementary Table S11).

In addition to the P39 lines, PTEN hypermethylation pattern was only observed in Kasumi-AZA-R (*p* = 0.001) but not Kasumi-1-DEC-R, which showed hypomethylation at most CpG loci (*p* = 0.003) (Table 1 and Supplementary Table S12). 

**Figure 4:**
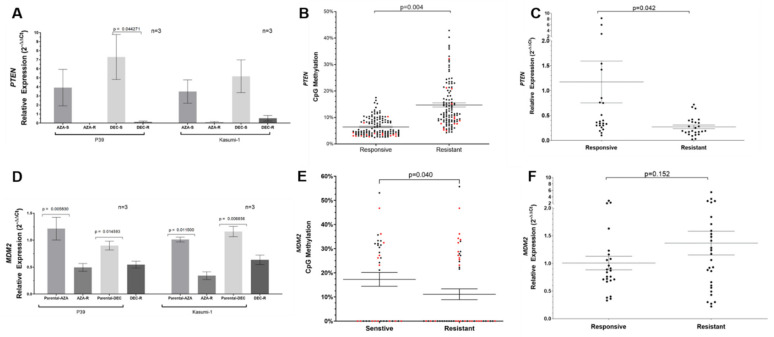

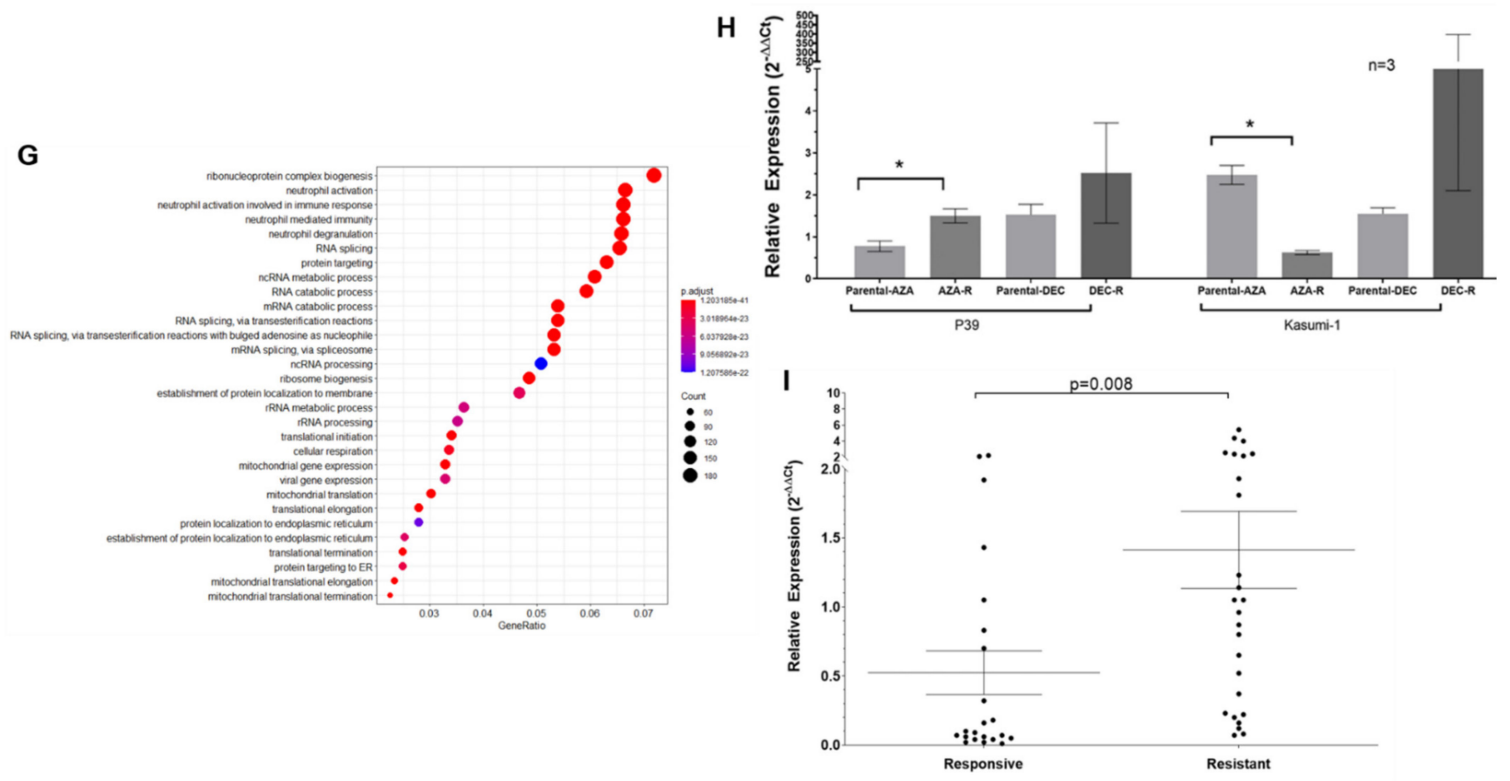
Association of PTEN and MDM2 methylation with mRNA expression. (**A**) Downregulation of PTEN by quantitative polymerase chain reaction (Q-PCR) in azacitidine and decitabine resistant cell lines; (**B**) Validation of PTEN DNA methylation in patients responsive or resistant to hypomethylating agents using pyrosequencing. Individual CpG methylation levels were displayed as individual data points with red data points (red dots) being the same loci (cg10041390) detected to be hypermethylated in 850k whole methylome microarray. The total number of dots denotes the number of CpG sites evaluated, where red dots d enote CpG sites with methylation changes and black dots denote flanking or additional CpG sites; (**C**) Downregulation of PTEN by Q-PCR in patients with myelodysplastic resistant to hypomethylating agents; (**D**) Expression of MDM2 in cell lines sensitive and resistant to azacitidine and decitabine; (**E**) Validation of MDM2 hypomethylation in patients with MDS resistant to hypomethylating agents by pyrosequencing. Individual CpG methylation levels were displayed as individual data points, with red data points (red dots) being the same loci (cg00614420) detected to be hypomethylated in 850k whole methylome microarray. The total number of dots denotes the number of CpG sites evaluated, where red dots denote CpG sites with methylation changes and black dots denote flanking or additional CpG sites; (**F**) Expression of MDM2 in patients with MDS responsive and resistant to hypomethylating agents; (**G**) Epi-transcriptomic profiling of P39 and azacitidine-resistant P39 (P39-AZA-R) cell line. Direct RNA sequencing of P39 cells and P39-AZA-R detected 5,340,619 differential methylated transcripts including the MDM2 processed transcript. Gene Ontogeny (GO) analysis demonstrated that the most enriched GO term comprised transcripts involved in ribonucleoprotein biogenesis and neutrophil functions (e-x denotes 10^x^); (**H**) Increased expression of the MDM2 truncated transcript was detected in P39-AZA-R but was downregulated in Kasumi-AZA-R compared with corresponding parental cells (*p* < 0.05 for both); (**I**) Increased expression of the MDM2 processed transcript in patients with myelodysplastic syndrome resistant to hypomethylating agents. Horizontal lines: mean ± standard error of mean (SEM); Unpaired *t*-tests were performed for statistical analysis, and two-tailed *p*-values < 0.05 were considered statistically significant; *: *p* < 0.05. P39 cells were treated separately with AZA and DEC at 1 µM for 48 h followed by immediate harvest for the assessment of methylation status and mRNA expression.

When all cell lines were studied for PTEN mRNA expression, all four HMA-resistant cell lines showed significant downregulation of PTEN compared with parental cells (Figure 4A).

For MDM2, three hypomethylated CpG loci cg00614420, cg09781971, and cg24703804 were examined in silico for the presence of CpG island or shores. Only cg00614420 and one adjacent CpG site mapping approximately 900 bp upstream to MDM2 were validated by pyrosequencing (Table 2 and Supplementary Table S13). Among all four HMA-resistant cell lines, hypomethylation of both CpG sites were observed with significance (all *p* < 0.05). When genomic MDM2 hypomethylation was correlated with MDM2 transcriptional activity, MDM2 mRNA expression level dropped in all resistant cell lines in comparison with their corresponding parental cells (*p* < 0.05, Figure 4D).

To explore alternative reasons for downregulated MDM2 expression, the somatic mutation profile was examined in the RNA sequencing data, with no MDM2 mutation detectable. The SNP distributions across known gene elements (5′-UTR, exon, intron, 3′-UTR and intergenic region) also showed no significant changes in HMA-resistant P39 sublines (Supplementary Table S14). On the other hand, a significant decrease in the number of fusion events was observed in both P39-AZA-R and P39-DEC-R, with only two and four fusions event remaining in P39-AZA-R and P39-DEC-R, respectively (Supplementary Table S15). The fusion genes observed in both sensitive and resistant lines included CLN6-CALML4, SRGAP2B-SRGAP2C, and CYFIP2-PLCG2.

### 3.5. Epi-Transcriptomic Profiling of P39 and P39-AZA-R

To explore potential mechanisms alternative to DNA methylation that might be involved in MDM2 downregulation in HMA-resistant P39 cells, whole epi-transcriptomic profiling was performed. Direct long-read sequencing of P39 and P39-AZA-R detected 5,340,619 differential methylated (m^6^A) RNA loci, and GO analysis predicted that the majority of dysregulated transcripts were associated with ribonucleoprotein biogenesis and neutrophil function (Figure 4G). Meanwhile, RNA splicing was the third most enriched pathway, and one of the MDM2 processed transcripts (ENST00000400501.2) was found to be significantly hypomethylated by 0.19-fold in P39 compared with P39-AZA-R (*p* = 1.12 × 10^−9^). This truncated transcript with predicted length of 406 bp was predicted to be non-protein coding, with the RING domain absent in comparison with normal MDM2. The processed MDM2 transcript was quantified in P39 and Kasumi-1 with their respective AZA-R and DEC-R sublines (Figure 4H). Results showed that the MDM2 processed transcript was upregulated in P39-AZA-R (*p* = 0.03), with a trend of upregulation also observed in P39-DEC-R and Kasumi-1-DEC-R (*p* = 0.33 and 0.18, respectively) in comparison with the parental lines. Interestingly, Kasumi-AZA-R showed a significant downregulation of the MDM2 processed transcript (*p* = 0.003). 

### 3.6. Validation in Primary MDS Patient Samples

The two important findings based on the multi-omic analyses in cell lines were validated in BM samples of patients with higher-risk MDS (Supplementary Table S16). Firstly, results from the HMA-resistant P39 sublines suggested that DNA methylation mediated silencing of PTEN. In BM samples, all CpG loci in PTEN including cg10041390 showed significantly higher methylation levels in HMA-resistant patients compared with HMA-responsive patients (Figure 4B, *p* = 0.004). Furthermore, PTEN expression was significantly downregulated in HMA-resistant patients compared with HMA-responsive patients (Figure 4C, *p* = 0.042). Secondly, in HMA-resistant sublines, hypomethylation of the CpG loci cg00614420 and one adjacent CpG site mapping approximately 900 bp upstream in MDM2 was observed. Similarly, pyrosequencing showed significant hypomethylation of the same CpG sites in BM samples from HMA-resistant patients compared with those observed in HMA-responsive patients (*p* = 0.040) (Figure 4E). In contrast to the observation in HMA-resistant cell lines, MDM2 expression was not significantly downregulated (*p* = 0.152) (Figure 4F) in HMA-resistant patients compared with HMA-responsive patients. The expression of the MDM2 processed transcript was also significantly increased in HMA-resistant patients compared with HMA-responsive patients (*p* = 0.008) (Figure 4I).

## 4. Discussion

In this study, transcriptome, methylome, and epitranscriptome were studied together to decipher the molecular mechanisms contributing to HMA resistance. In the MDS/secondary AML cell line P39, we showed consistent downregulation of PTEN and MDM2 and increased expression of a truncated MDM2 transcript during HMA resistance. However, in the comparator AML line Kasaumi-1, which contained RUNX1-RUNX1T1, such changes were not consistently observed. P39 was selected for experimentation, despite its possible contamination with HL-60, an AML cell line [30,31], because it is the most widely employed cell line reminiscent of the biology of MDS used to evaluate responses to hypomethylating agents [32,33]. With the exception CYFIP2-PLCG2, which was present in the P39 cell line in this study, other fusion genes reported in HL-60 were not observed in the P39 cell line that we acquired [34]. 

DNA hypermethylation leading to downregulated expression of the tumor suppressor PTEN was observed in cell lines and in primary patient specimens. These changes were predicted to impede the tumor suppressive function of PTEN in the PI3K-AKT-MDM2 signaling pathway [35,36,37]. In addition, PTEN can function in a phosphatase-independent manner, and this includes maintenance of genome stability mediated by mono-ubiquitination of nuclear PTEN and interaction with APC/CDH1 to regulate cell proliferation [37]. Reduced expression of PTEN has been reported in high-risk MDS and is related to a constitutive activation of AKT via phosphorylation [37]. A single knockout of PTEN in a murine model was insufficient to produce an MDS phenotype, but PTEN deficiency in combination with SHIP knockout resulted in a severe MDS phenotype. The levels of PTEN and SHIP are the key factors, as the two phosphatases share the same substrate PIP_3_ [38]. 

In this study, we demonstrated that downregulation of PTEN in HMA resistance induced in vitro was associated with DNA methylation. Interestingly, while PTEN was not methylated in P39, a low level of DNA methylation was sufficient to suppress PTEN transcription in P39-AZA-R and P39-DEC-R. Another mechanism that may be involved in the profound PTEN downregulation observed in addition to DNA methylation might involve the feedback loop of PTEN and p53. A positive feedback mechanism was proposed, with PTEN essential for p53-dependent senescence by maintaining p53 stability, and the PTEN-p53 tetramer enhancing p53 DNA binding and transcriptional activity [35,39,40].

In hematologic malignancies, murine models with PTEN deletions have been observed in association with myeloproliferative neoplasm (MPN), chronic myeloid leukemia (CML), and MDS. However, PTEN downregulation due to genetic or epigenetic alteration has not been previously described in AML [37,41]. In contrast to P39, PTEN remained mostly unmethylated in HMA-sensitive and HMA-resistant Kasumi-1, with only one CpG locus being methylated in Kasumi-DEC-R. In Kasumi-1, PTEN downregulation during AZA/DEC resistance might be related to RUNX1-RUNX1T1 suppression of PTEN via epigenetic silencing of the tumor-suppressive miR-193a [42]. 

Unlike PTEN that is a positive regulator of p53, the MDM2 protein is a RING finger E3 ubiquitin ligase commonly known to suppress p53 [43,44]. It hetero-dimerizes with MDM4 (also known as MDMX) to enhance p53 mono- or poly-ubiquitination under cellular stress. This facilitates p53 nuclear-to-cytoplasmic translocation, with mono-ubiquitination of p53 inhibiting transcriptional activity and poly-ubiquitination enhancing p53 degradation in normal cells [43,44]. In this study, MDM2 was downregulated in all HMA-resistant cell lines despite significant DNA hypomethylation. On the other hand, the MDM2 splicing machinery was altered as evidenced by the upregulation of a truncated MDM2 transcript. This suggested that the modulation of MDM2 observed during HMA resistance was dependent on RNA methylation rather than DNA methylation. Since the truncated MDM2 transcript observed does not possess an open reading frame or the RING domain responsible for p53 binding, the biologic consequence was downregulation of MDM2 function [45]. Therefore, MDM2 is functionally downregulated with the production of this truncated MDM2 transcript. Multiple studies have reported MDM2 as a bi-functional regulator of p53, it and may be involved in a p53-MDM2 feedback loop [46,47,48,49]. In situations where MDM2 functions as a TP53 enhancer, MDM2 stimulates TP53 mRNA translation while suppressing its poly-ubiquitination and proteasomal degradation. As expected, MDM2 also promotes degradation of mutated p53 in a similar manner as wild-type p53 in vivo, and functions as tumor-suppressor to prevent solid tumor formation [46].

MDM2 also possesses multiple function in hematological neoplasms. Firstly, MDM2 is anti-apoptotic, restoring erythroid progenitors from p53-mediated apoptosis. Double knockout of MDM2 in murine models compromised erythropoiesis leading to a phenotype similar to MDS with del(5q), which was lethal during postnatal hematopoiesis [43,44]. In the context of MDM2 dysregulation, MDM2 mutation at the promoter (MDM2^SNP309^) has been described in MDS patients [50]. Co-occurrence of MDM2^SNP309^ with the TP53 R72P mutation adversely affected overall survival and progression-free survival in non-del(5q) MDS but not isolated del(5q) MDS [50]. Similar genotypic analyses in AML showed worse outcomes in AML patients with MDM2^SNP309^ that was associated with increased MDM2 expression [51]. In contrast, a concomitant downregulation of MDM2 with p53 was observed in MDS with del(5q), with MDM2 responsible for enhancing p53 auto-ubiquitination and degradation [52]. In addition, treatment with lenalidomide induced MDM2 expression that accelerated p53 degradation, suggesting that the function of MDM2 in MDS was complex [52]. Therefore, the role of MDM2 during in myeloid development remains to be better defined.

**Figure 5:**
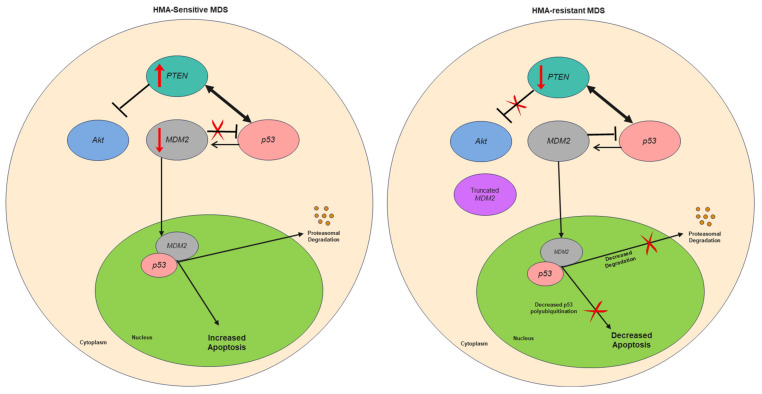
Proposed molecular mechanism of resistance to hypomethylating agents involving PTEN and MDM2. During resistance to hypomethylating agents, further suppression of PTEN is associated with CpG DNA hypermethylation. RNA M6A hypomethylation led to unexpected alternative splicing, which resulted in up-regulation of truncated MDM2 mRNA as non-coding transcript with only the 3′ untranslated region. PTEN functions as a tumor suppressor gene and inhibits PI3K/Akt signaling. This inhibition is lost upon DNA hypermethylation of PTEN during HMA-resistance. On the other hand, a shift in the function of MDM2 could be associated with HMA-resistance, with MDM2 initially being an oncoprotein conventionally known to negatively regulate p53. During HMA resistance, MDM2 is switched into a p53 enhancer, but the expression of MDM2 was suppressed due to RNA hypomethylation and altered MDM2 splicing. Therefore, PTEN and MDM2 silencing collectively result in dysregulated p53.

Given the fact that MDM2 expression was downregulated in all four HMA-resistant sublines in this study and that P39 and Kasumi-1 did not harbor chromosome 5q deletion, both the tumor-suppressive role independent of 5q deletion and the conventional proto-oncogenic role of MDM2 observed in del(5q) MDS should be considered [53]. Due to the fact that the functional impact of MDM2 on the common myeloid progenitor or granulocyte monocyte progenitor is unknown, we cannot exclude a potential oncogenic role of MDM2 in cell differentiation and within the bone marrow environment [54]. In the context of cell cycle regulation and apoptosis, we hypothesize that the HMA-resistant cells evade apoptosis not only by PTEN suppression but also by MDM2 downregulation, with both PTEN and MDM2 functioning as tumor suppressors. Taking into account the oncogenic role of MDM2 reported in del(5q) MDS and AML, we further propose a functional switch of MDM2 from oncogenic to tumor suppressive during HMA resistance, with loss of MDM2 being attributed to RNA hypomethylation (Figure 5) [50,51] In addition, the truncated MDM2 observed in our study only retains the 3′ UTR and is in-keeping with a long non-coding RNA without open reading frame [55]. It has been shown that the iron-binding protein IRP2 binds to the 3′UTR of MDM2, which results in stabilization of the MDM2 transcript [56]. We postulate that there is a competitive binding between normal MDM2 and the truncated MDM2 for IRP2, resulting in MDM2 mRNA instability and degradation during HMA resistance. The downregulation of MDM2 was clearly observed on our cell line models despite being not too noticeable in patients. This supports our proposition that MDM2 switched from an oncogene to tumor suppressor gene during treatment with HMA with MDM2 downregulation contributing to HMA resistance.

## 5. Conclusions

In conclusion, this study demonstrated that DNA methylation-mediated downregulation of PTEN and epi-transcriptional silencing of MDM2 in HMA-resistant cell line are involved in p53 dysregulation via PI3K/Akt/mTOR signaling. Moreover, this study demonstrated the effect of HMA on RNA methylation contributing to alternative splicing in MDM2. We propose a novel mechanism of p53 dysregulation that involves a functional switch of MDM2 during HMA resistance. Further validation of this model, particularly the role of RNA methylation in HMA-resistance, is warranted. Furthermore, our results also showed oncogenic addiction to the dysregulated PI3K/Akt/mTOR signaling, PTEN and MDM2 during HMA resistance. These may serve as a target for therapeutic modulation by small molecules [57,58,59].

## Figures and Tables

**Table 1:** PTEN methylation of cell lines sensitive to and resistant to hypomethylating agents.

Cell Line *	CpG 1 (%)	CpG 2 (%)	CpG 3 (%)	CpG 4 (%)	CpG 5 (%)	CpG 6# (%)	CpG 7 (%)	CpG 8 (%)	CpG 9 (%)	Mean (%)
Parental P39 sensitive to AZA **	5.95	7.78	7.41	11.91	7.23	3.96	3.54	6.04	14.1	7.55
Parental P39 sensitive to DEC **	4.91	6.5	6.91	12.37	7.57	4.39	3.8	6.7	13.7	7.43
P39-AZA-R	4.87	7.46	6.82	13.07	8.69	4.67	5.01	7.22	15.16	8.11
P39-DEC-R	5.47	7.88	8.07	12.93	8.4	4.35	3.94	6.87	15.16	8.12
Parental Kasumi-1 sensitive to AZA **	5.86	7.98	5.61	11.57	7.56	4.53	4.22	6.09	13.48	7.43
Parental Kasumi-1 sensitive to DEC **	4.86	7.69	5.35	11.75	6.66	4.31	4.54	6.89	14.45	7.39
Kasumi-1-AZA-R	6.56	8.52	7.35	15.41	7.77	5.67	6.53	8.21	18.82	9.43
Kasumi-1-DEC-R	4.61	7.26	5.74	11.18	6.07	4.19	3.74	5.52	12.34	6.74

* P39, Kasumi-1 and their derivative cell lines resistant to either azacitidine or decitabine were analyzed for PTEN methylation by pyrosequencing. ** The sensitive parental P39 and Kasumi-1 cells were treated separately with AZA and DEC at 1 µM for 48 h followed by immediate harvest for the assessment. #: The sixth CpG site analyzed corresponded to the same loci (cg10041390) detected to be hypermethylated in 850k whole methylome microarray. P39-AZA-S: Azacitidine-sensitive P39 cell line; P39-DEC-S: Decitabine-sensitive P39 cell line; P39-AZA-R: Azacitidine-resistant P39 cell line; P39-DEC-R: Decitabine-resistant P39 cell line; Kasumi-1-AZA-S: Azacitidine-sensitive Kasumi-1 cell line; Kasumi-1-DEC-S: Decitabine-sensitive Kasumi-1 cell line; Kasumi-AZA-R: Azacitidine-resistant Kasumi-1 cell line; Kasumi-1-DEC-R: Decitabine-resistant Kasumi-1 cell line.

**Table 2:** MDM2 methylation of cell lines sensitive to and resistant to hypomethylating agents.

Cell Line *	CpG 1 (%)	CpG 2 (%) #	Mean (%)
Parental P39 sensitive to AZA **	45.92	28.05	36.99
Parental P39 sensitive to DEC **	56.31	32.24	44.28
P39-AZA-R	26.37	16.77	21.57
P39-DEC-R	31.44	21.71	26.58
Parental Kasumi-1 sensitive to AZA **	57.97	35.89	46.93
Parental Kasumi-1 sensitive to DEC **	66.45	42.91	54.68
Kasumi-1-AZA-R	37.49	19.56	28.53
Kasumi-1-DEC-R	44.07	26.95	35.51

* P39, Kasumi-1 and their derivative cell lines resistant to either azacitidine or decitabine were analyzed for MDM2 methylation by pyrosequencing. ** The sensitive parental P39 and Kasumi-1 cells were treated separately with AZA and DEC at 1 µM for 48 h followed by immediate harvest for the assessment. # The second CpG analyzed by pyrosequencing was the same loci (cg00614420) detected hypomethylated in 850k whole methylome microarray. P39-AZA-S: Azacitidine-sensitive P39 cell line; P39-DEC-S: Decitabine-sensitive P39 cell line; P39-AZA-R: Azacitidine-resistant P39 cell line; P39-DEC-R: Decitabine-resistant P39 cell line; Kasumi-1-AZA-S: Azacitidine-sensitive Kasumi-1 cell line; Kasumi-1-DEC-S: Decitabine-sensitive Kasumi-1 cell line; Kasumi-AZA-R: Azacitidine-resistant Kasumi-1 cell line; Kasumi-1-DEC-R: Decitabine-resistant Kasumi-1 cell line.

## Data Availability

All high-throughput sequencing data that supported the findings of this study were deposited in the Gene Expression Omnibus (GEO) with the accession code (GSE165188). For specific dataset, the accession numbers are GSE165185 for methylation microarray data, GSE165187 for RNA transcriptomic data, and GSE165064 for native RNA sequencing data for epi-transcriptomic analysis. Other relevant data supporting the key findings of this study are available within the article or upon request to the corresponding author.

## Supplementary Materials

The following supporting information can be downloaded at: https://www.mdpi.com/article/10.3390/ijms23105670/s1.
