# Supplementary material for: Epigenetic Silencing of PTEN and Epi-Transcriptional Silencing of MDM2 Underlied Progression to Secondary Acute Myeloid Leukemia in Myelodysplastic Syndrome Treated with Hypomethylating Agents
Source: Int J Mol Sci. 2022 May 18;23(10):5670. doi: 10.3390/ijms23105670 (PMC9144309; doi:10.3390/ijms23105670)
Supplement: Supplementary file 1 [file ijms-23-05670-s001.zip › Figure S1.pdf]

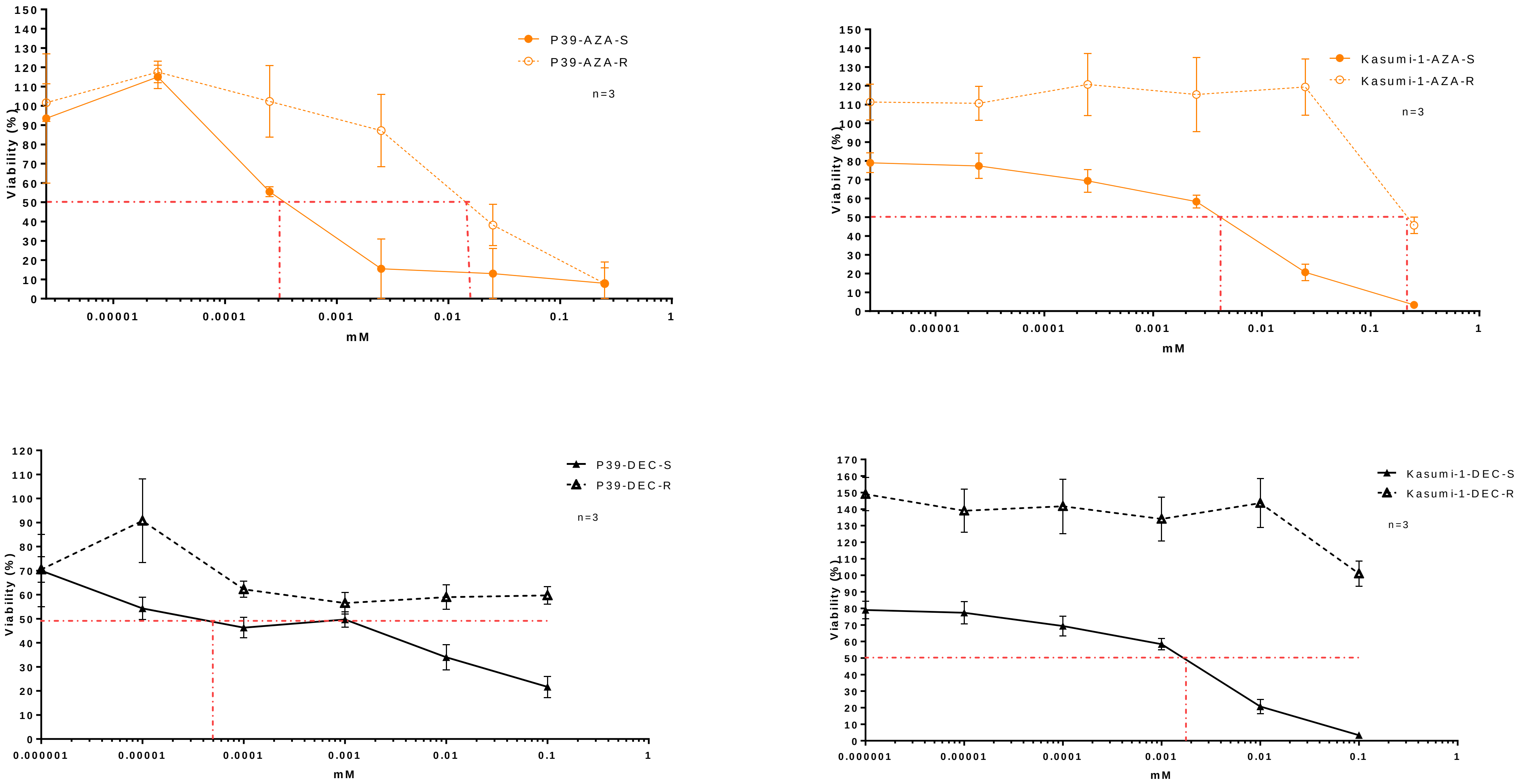

Figure S1. IC_{50} of hypomethylating agent-resistant cell lines. The IC_{50} of P39 and Kasumi-1 cells with resistance to azacitidine (AZA) and decitabine (DEC) were determined and compared with parental cells by transient culture with increasing doses of AZA and DEC for 48 hours. Cell viability was measured by presto blue assay and hypomethylating agent (HMA)-resistant cells with resistance to at least 500nM of AZA or DEC were harvested for further evaluation. P39-AZA-S: Azacitidine-sensitive parental P39 cells; P39-DEC-S: decitabine-sensitive parental P39 cells P39-AZA-R: Azacitidine-resistant P39 cells; P39-DEC-R: decitabine-resistant P39 cells; Kasumi-1-AZA-S: Azacitidine-sensitive parental Kasumi-1 cells; Kasumi-1-DEC-S: decitabine-sensitive parental Kasumi-1 cells; Kasumi-1-AZA-R: Azacitidine-resistant Kasumi-1 cells; Kasumi-1-DEC-R: decitabine-resistant Kasumi-1 cells
