# Supplementary material for: Epigenetic Silencing of PTEN and Epi-Transcriptional Silencing of MDM2 Underlied Progression to Secondary Acute Myeloid Leukemia in Myelodysplastic Syndrome Treated with Hypomethylating Agents
Source: Int J Mol Sci. 2022 May 18;23(10):5670. doi: 10.3390/ijms23105670 (PMC9144309; doi:10.3390/ijms23105670)
Supplement: Supplementary file 1 [file ijms-23-05670-s001.zip › Figure S2.pdf]

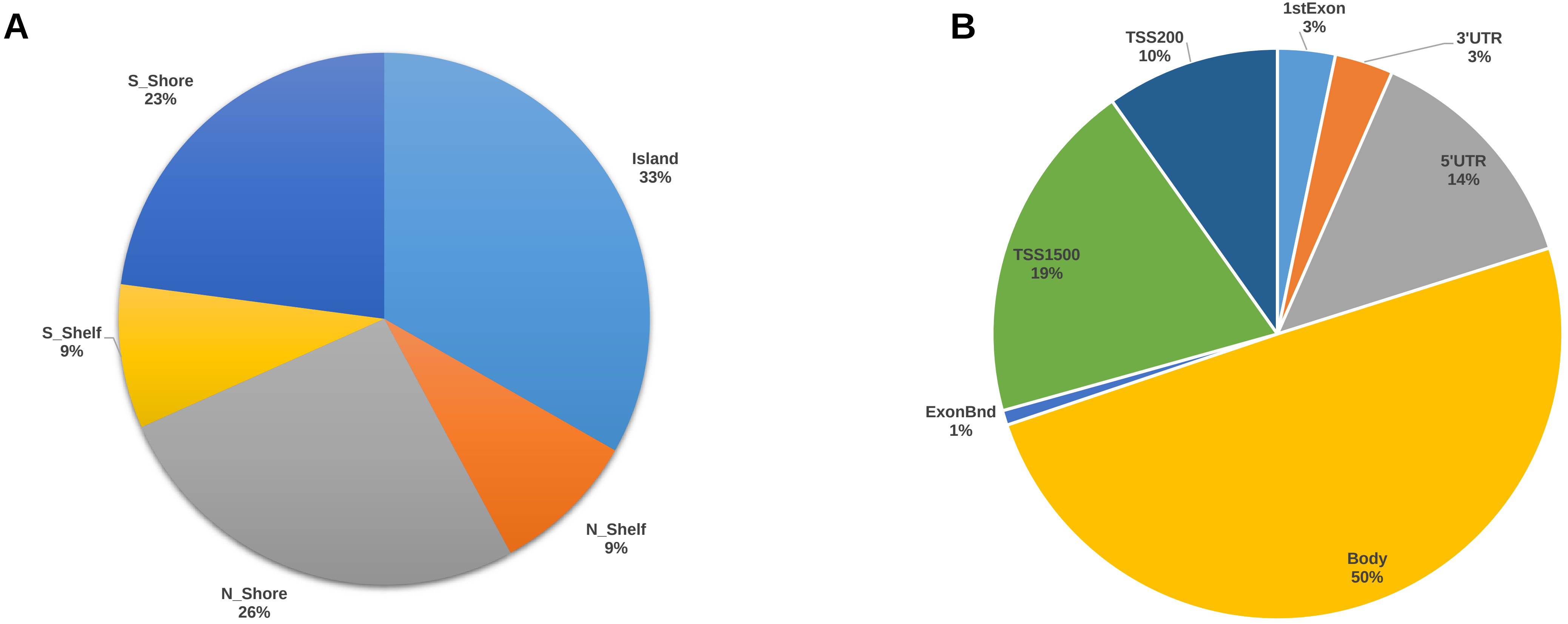

Figure S2. Classification of differentially methylated regions (DMRs) based on **A:** relative location to CpG Island; and **B:** relative location of known University of California Santa Cruz (USC) RefGene structures. HMA: hypomethylating agent; UTR: untranslated region; TSS: transcription start site; TSS1500: transcription start site 1500 base-pairs upstream; TSS200: transcription start site 200 base-pairs upstream
