# Supplementary material for: Epigenetic Silencing of PTEN and Epi-Transcriptional Silencing of MDM2 Underlied Progression to Secondary Acute Myeloid Leukemia in Myelodysplastic Syndrome Treated with Hypomethylating Agents
Source: Int J Mol Sci. 2022 May 18;23(10):5670. doi: 10.3390/ijms23105670 (PMC9144309; doi:10.3390/ijms23105670)
Supplement: Supplementary file 1 [file ijms-23-05670-s001.zip › Figure S3.pdf]

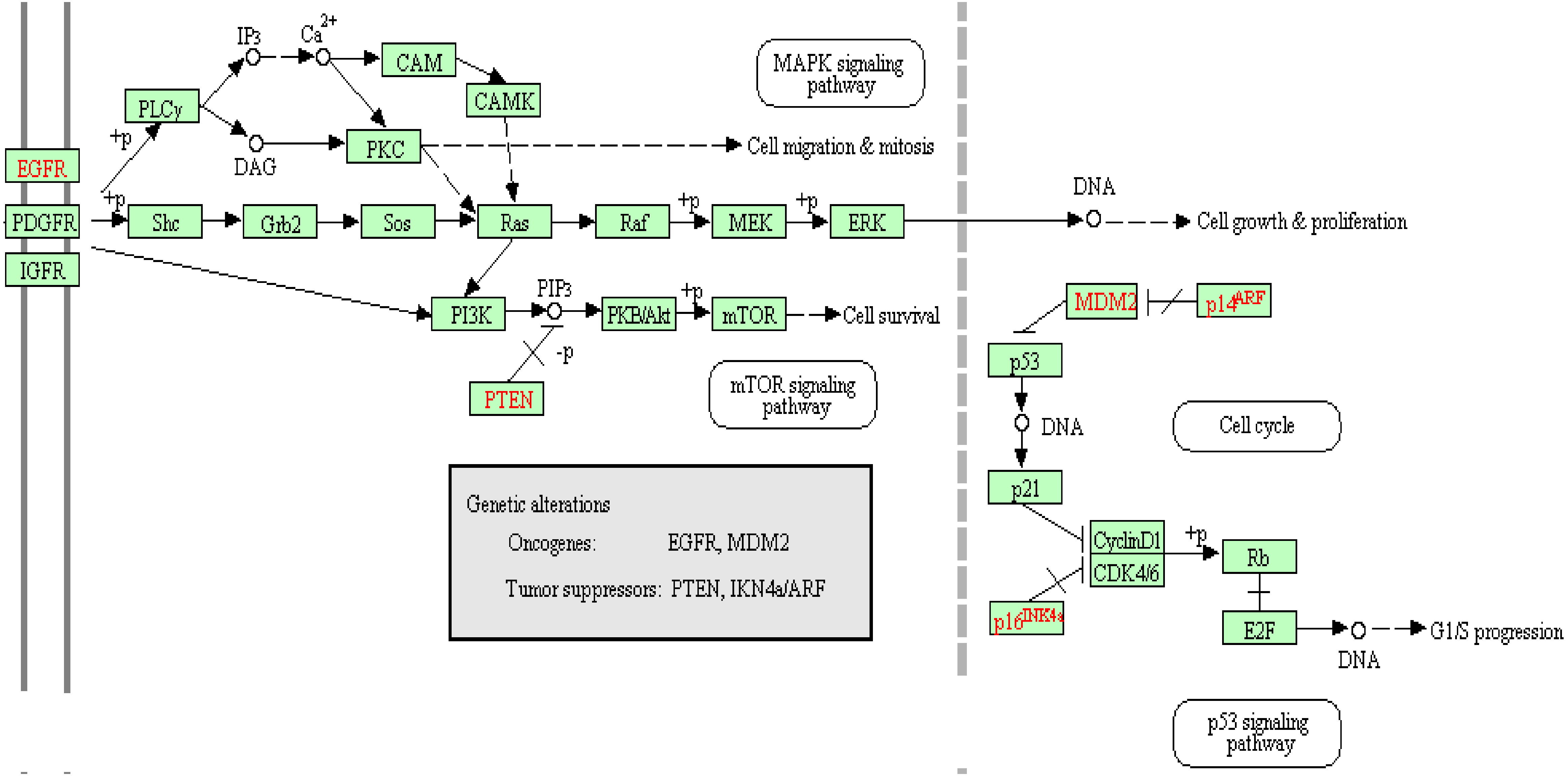

Figure S3. Functional prediction of Gene Ontogeny (GO) enriched gene list by Kyoto Encyclopedia of Genes and Genomes (KEGG) analysis with differential methylation signature in hypomethylating agent-resistant cell lines. Selected differentially methylated genes submitted for functional prediction predicted that the mTOR signaling pathway involving *PTEN* and *MDM2* were involved in resistance to hypomethylating agents. Other genes predicted involved in such pathway included *EGFR*, *p16INK4a* and *p14ARF* that were directly or indirectly regulated by *PTEN* and *MDM2*
