# Supplementary material for: Epigenetic Silencing of PTEN and Epi-Transcriptional Silencing of MDM2 Underlied Progression to Secondary Acute Myeloid Leukemia in Myelodysplastic Syndrome Treated with Hypomethylating Agents
Source: Int J Mol Sci. 2022 May 18;23(10):5670. doi: 10.3390/ijms23105670 (PMC9144309; doi:10.3390/ijms23105670)
Supplement: Supplementary file 1 [file ijms-23-05670-s001.zip › Figure S4.pdf]

P39-AZA-R

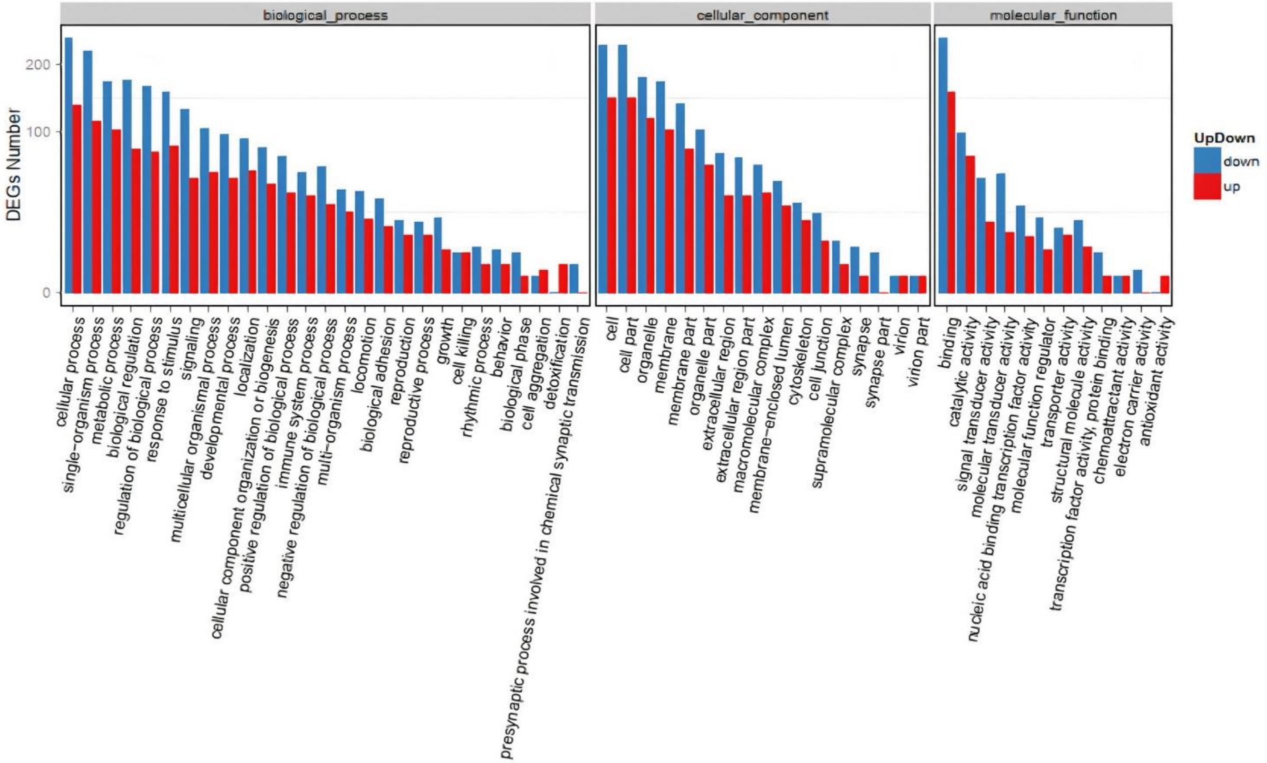

P39-DEC-R

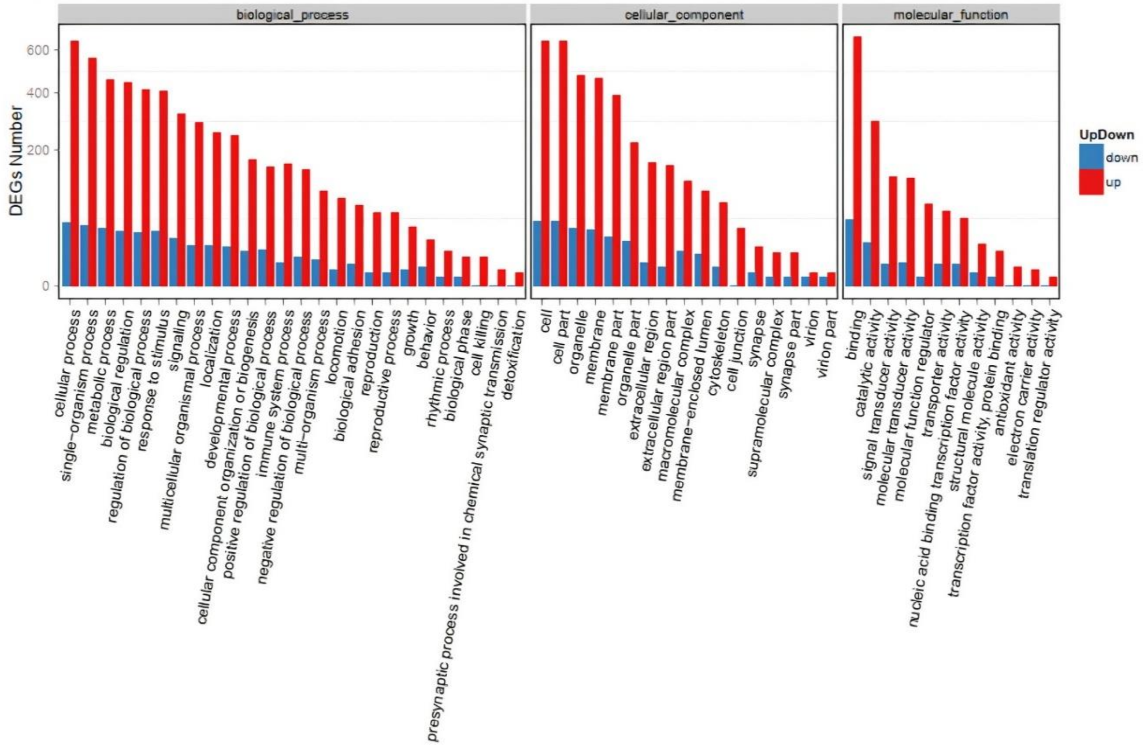

Figure S4. GO prediction for pathway and functional roles of differentially expressed genes (DEGs) in cell lines. Functional prediction by GO analysis of differentially expressed genes in hypomethylating agent (HMA)-resistant P39 cell line compared with the corresponding HMA sensitive P39 cell lines was performed separately for azacitidine-resistant P39 cell line (P39-AZA-R) and decitabine-resistant P39 cell line (P39-DEC-R). Both cell lines had dysregulated PI3K/Akt signaling that were predicted to be mediated by Platelet Derived Growth Factor Subunit A (PDGFA) and Endothelia PAS domain-containing protein 1 (EPAS1) upon correlation with DNA methylation patterns. Both cell lines also showed similar dysregulation in cellular processes. GO: Gene Ontogeny.
