# Supplementary material for: Epigenetic Silencing of PTEN and Epi-Transcriptional Silencing of MDM2 Underlied Progression to Secondary Acute Myeloid Leukemia in Myelodysplastic Syndrome Treated with Hypomethylating Agents
Source: Int J Mol Sci. 2022 May 18;23(10):5670. doi: 10.3390/ijms23105670 (PMC9144309; doi:10.3390/ijms23105670)
Supplement: Supplementary file 1 [file ijms-23-05670-s001.zip › Figure S5.pdf]

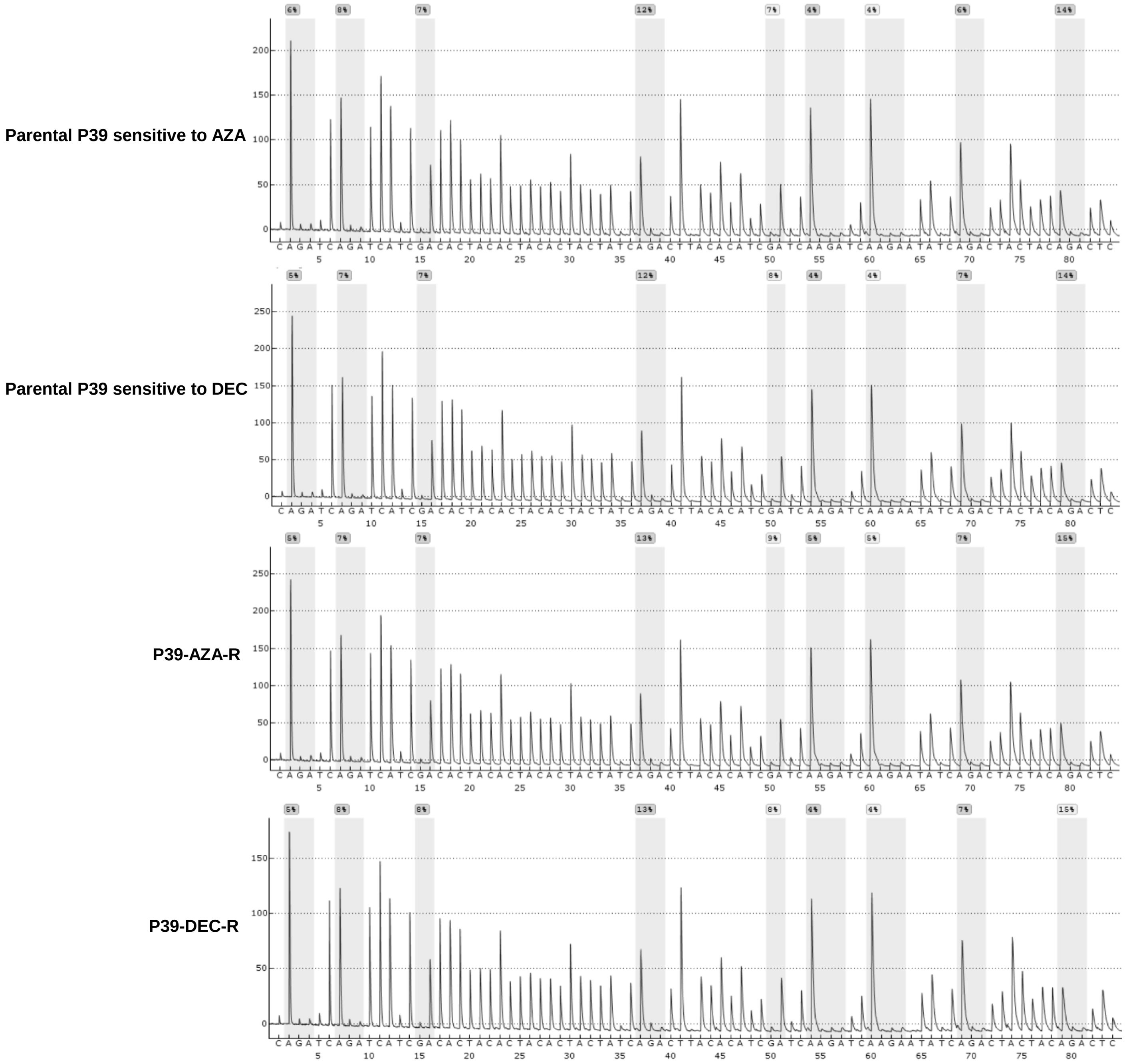

Figure S5. Pyrosequencing of *PTEN* in P39 cell lines. Pyrograms of hypermethylated CpG upstream to *PTEN*. The fourth CpG analyzed by pyrosequencing was the same loci (cg10041390) detected to be hypermethylated in 850k whole methylome microarray. AZA: Azacitidine; DEC: decitabine; P39-AZA-R: Azacitidine-resistant P39 cell line; P39-DEC-R: decitabine-resistant P39 cell line. The sensitive parental P39 cells were treated separately with AZA and DEC at 1 μM for 48 hours followed by immediate harvest for the assessment
