# Supplementary material for: Epigenetic Silencing of PTEN and Epi-Transcriptional Silencing of MDM2 Underlied Progression to Secondary Acute Myeloid Leukemia in Myelodysplastic Syndrome Treated with Hypomethylating Agents
Source: Int J Mol Sci. 2022 May 18;23(10):5670. doi: 10.3390/ijms23105670 (PMC9144309; doi:10.3390/ijms23105670)
Supplement: Supplementary file 1 [file ijms-23-05670-s001.zip › Figure S7.pdf]

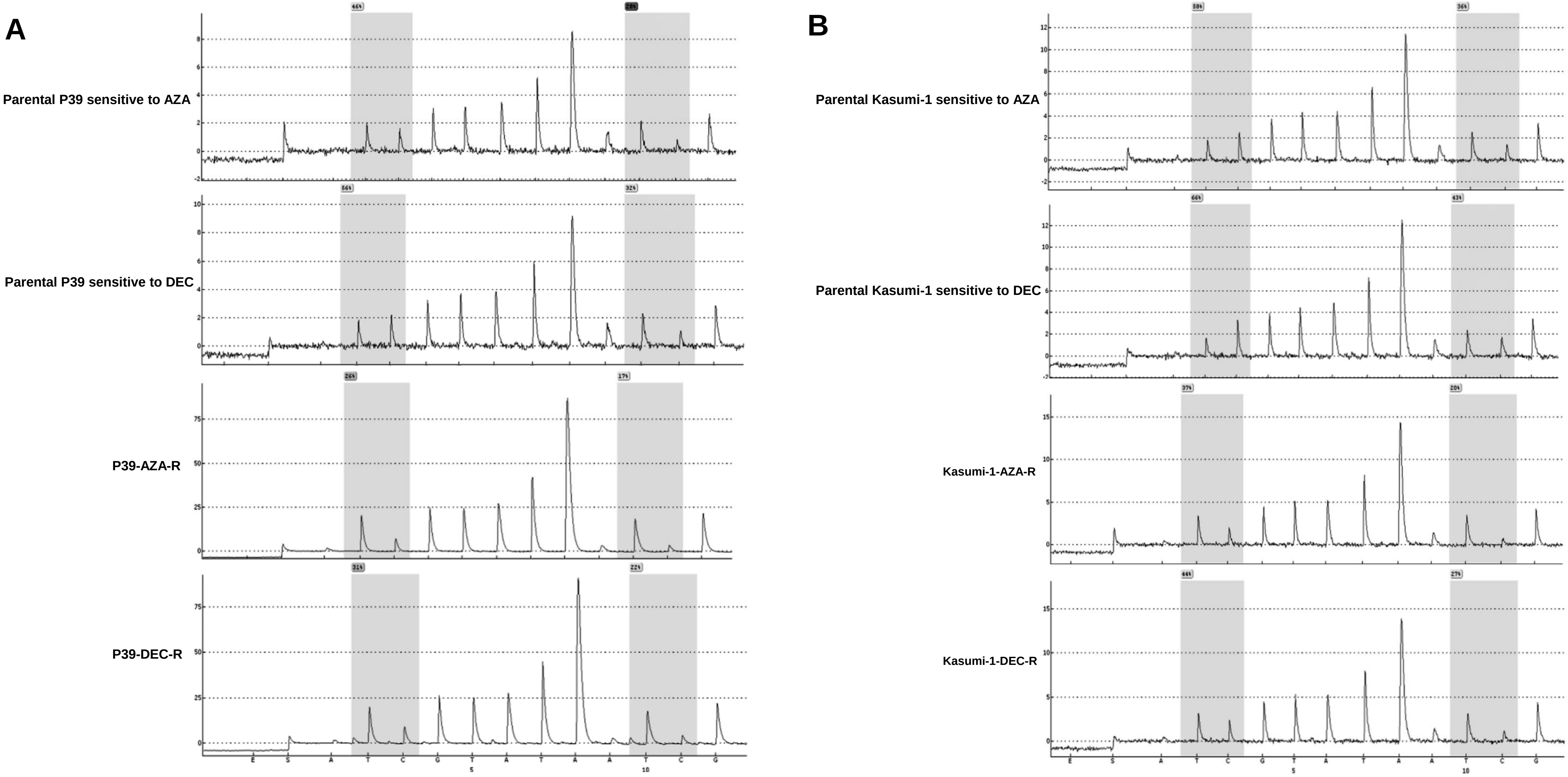

Figure S7. Pyrosequencing of *MDM2* in P39 and Kasumi-1 cell lines. **A:** Pyrograms of hypermethylated CpG upstream to *MDM2* in the azacitidine and decitabine-sensitive parental P39 cell lines, P39-AZA-R, and P39-DEC-R. The second CpG analyzed by pyrosequencing was the same loci (cg00614420) detected to be hypermethylated in 850k whole methylome microarray; **B:** Pyrograms of hypermethylated CpG upstream to *MDM2* in the azacitidine and decitabine sensitive parental Kasumi-1 cell lines, Kasumi-1-AZA-R, and Kasumi-1-DEC-R. The second CpG analyzed by pyrosequencing was the same loci (cg00614420) detected to be hypermethylated in 850k whole methylome microarray. AZA: azacitidine; DEC: decitabine; P39-AZA-R: Azacitidine-resistant P39 cell line; P39-DEC-R: decitabine-resistant P39 cells line; Kasumi-1-AZA-R: Azacitidine-resistant Kasumi-1 cell line; Kasumi-1-DEC-R: decitabine-resistant Kasumi-1 cell line. The sensitive parental P39 and Kasumi-1 cells were treated separately with AZA and DEC at 1 μM for 48 hours followed by immediate harvest for the assessment
