# Supplementary material for: Epigenetic Silencing of PTEN and Epi-Transcriptional Silencing of MDM2 Underlied Progression to Secondary Acute Myeloid Leukemia in Myelodysplastic Syndrome Treated with Hypomethylating Agents
Source: Int J Mol Sci. 2022 May 18;23(10):5670. doi: 10.3390/ijms23105670 (PMC9144309; doi:10.3390/ijms23105670)
Supplement: Supplementary file 1 [file ijms-23-05670-s001.zip › Figure S8.pdf]

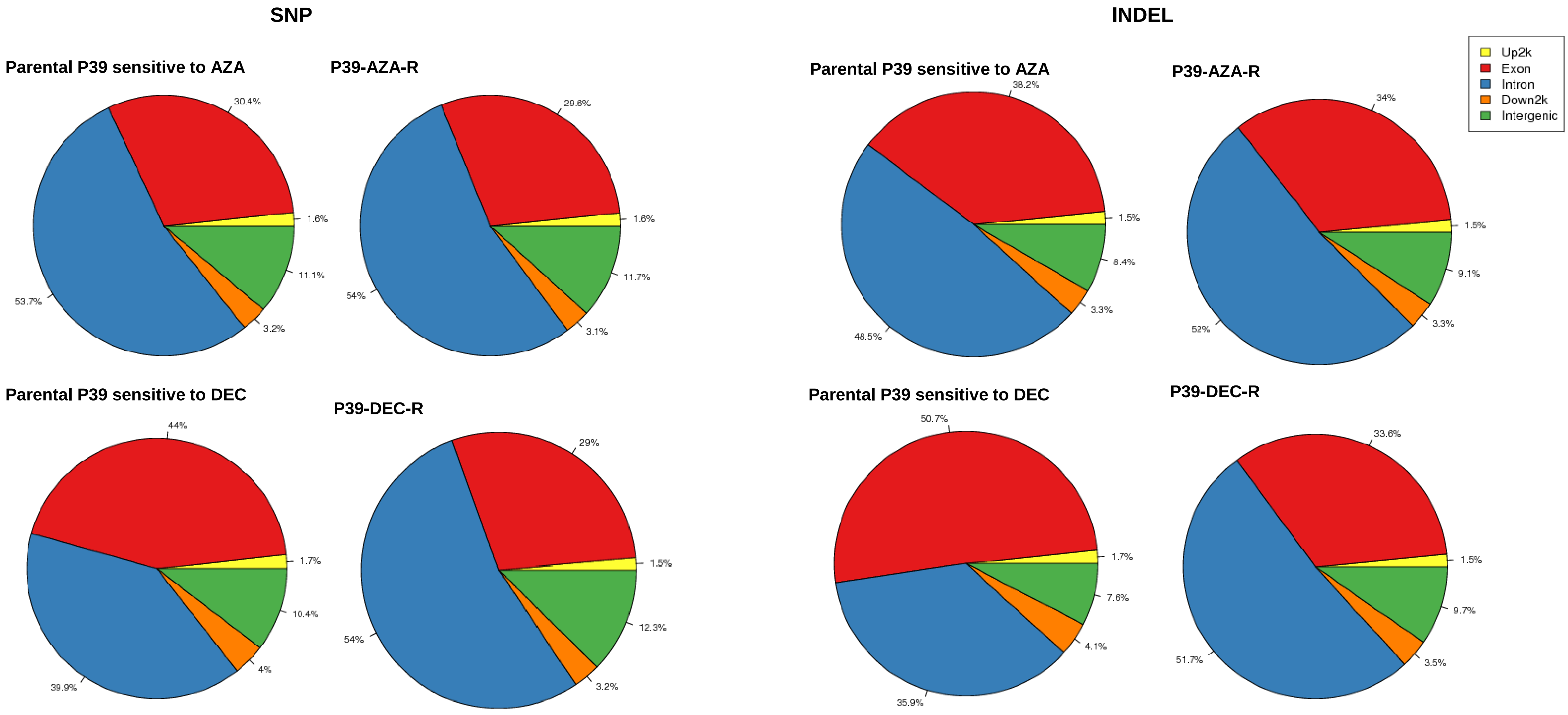

Figure S8. Distribution of mutations detected across different gene elements. Mutation profiling of parental P39 cells sensitive to azacitidine and decitabine were compared with azacitidine-resistant and decitabine-resistant P39 cells which somatic SNP and INDEL were called and classified based on localization to different gene elements. Majority of distributions were similar across all cell lines. SNP: single nucleotide polymorphism; INDEL: insertion/deletion; AZA: azacitidine; DEC: decitabine; P39-AZA-R: Azacitidine-resistant P39 cell line; P39-DEC-R: decitabine-resistant P39 cell line. The sensitive parental P39 cells were treated separately with AZA and DEC at 1 μM for 48 hours followed by immediate harvest for the assessment
