# Supplementary material for: Epigenetic Silencing of PTEN and Epi-Transcriptional Silencing of MDM2 Underlied Progression to Secondary Acute Myeloid Leukemia in Myelodysplastic Syndrome Treated with Hypomethylating Agents
Source: Int J Mol Sci. 2022 May 18;23(10):5670. doi: 10.3390/ijms23105670 (PMC9144309; doi:10.3390/ijms23105670)
Supplement: Supplementary file 1 [file ijms-23-05670-s001.zip › Figure S9.pdf]

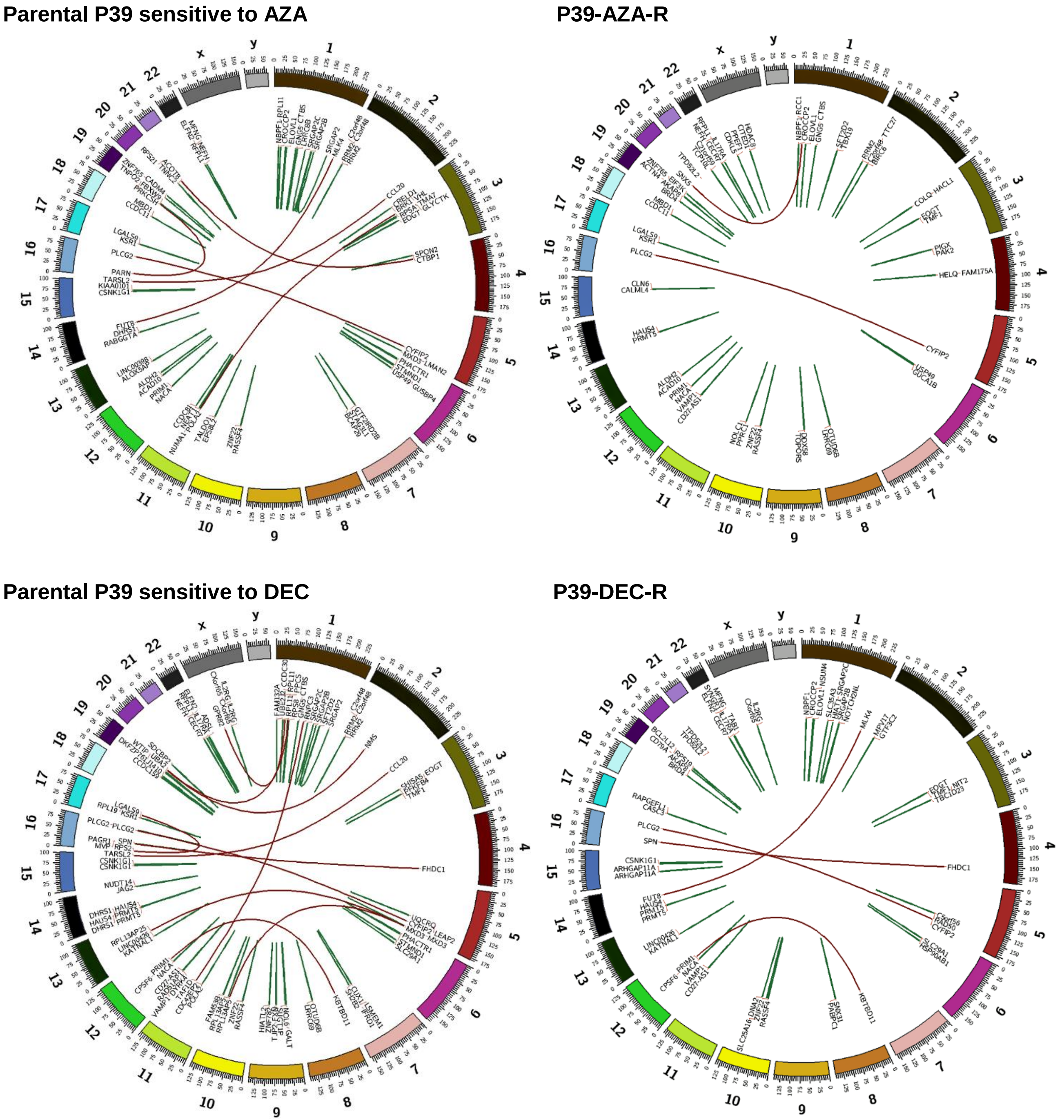

Figure S9. Gene fusion events in P39 cell lines resistant to hypomethylating agents compared with those sensitive to hypomethylating agents. Gene fusions were detected in P39 cell lines and visualized with Circos plots. There were more fusion events detect in both P39-AZA-R and P39-DEC-R in comparison with corresponding azacitidine and decitabine-sensitive parental cell lines. Among all fusion events detected, *CYFIP2-PLCG2* was the only fusion transcript detected in both P39-AZA-R and P39-DEC-R and it was the dominant fusion gene observed in both hypomethylating agent-resistant cell line. P39-AZA-R: Azacitidine-resistant P39 cell line; P39-DEC-R: decitabine-resistant P39 cell line. The sensitive parental P39 cells were treated separately with AZA and DEC at 1 μM for 48 hours followed by immediate harvest for the assessment
