# Supplementary material for: Epigenetic Silencing of PTEN and Epi-Transcriptional Silencing of MDM2 Underlied Progression to Secondary Acute Myeloid Leukemia in Myelodysplastic Syndrome Treated with Hypomethylating Agents
Source: Int J Mol Sci. 2022 May 18;23(10):5670. doi: 10.3390/ijms23105670 (PMC9144309; doi:10.3390/ijms23105670)
Supplement: Supplementary file 1 [file ijms-23-05670-s001.zip › Table S2.pdf]

Table S2. Genomic distribution of differentially methylated regions (DMRs).

A: Genomic distribution of differentially methylated regions (DMRs) between hypomethylating agent (HMA)-sensitive and -resistant cells with respect to CpG density.

CpG context	UCSC CpG prediction*
Island	6689
Upstream to Shelf	1786
Upstream to Shore	5275
Downstream to Shelf	1762
Downstream to Shore	4612
Others	2
Total	20126

*: Summary of DMR location relative to CpG islands DMRs are summarized regardless of hypermethylation or hypomethylation.
USCS: University of California Santa Cruz Genomics institute.

B: Genomic distribution of differentially methylated regions (DMRs) between hypomethylating agent (HMA)-sensitive and -resistant cells with respect to gene context.

Gene Context	UCSC Gene prediction*
First Exon	1196
3'UTR	1209
5'UTR	4950
Body	18154
Exon boundaries	307
TSS 1500bp upstream	7121
TSS 200bp upstream	3591
Others	2
Grand Total	36530

*: Summary of DMR relative location to known USCS RefGene structures are summarized. DMRs are summarized regardless of hypermethylation or hypomethylation but majority of hypermethylated DMRs were localized to upstream promoter or first exon of gene body.
USCS: University of California Santa Cruz Genomics institute; UTR: untranslated region; TSS: transcription start site; bp: base-pairs.
