# Supplementary material for: Epigenetic Silencing of PTEN and Epi-Transcriptional Silencing of MDM2 Underlied Progression to Secondary Acute Myeloid Leukemia in Myelodysplastic Syndrome Treated with Hypomethylating Agents
Source: Int J Mol Sci. 2022 May 18;23(10):5670. doi: 10.3390/ijms23105670 (PMC9144309; doi:10.3390/ijms23105670)
Supplement: Supplementary file 1 [file Figure S6.pdf › Figure S6.pdf]

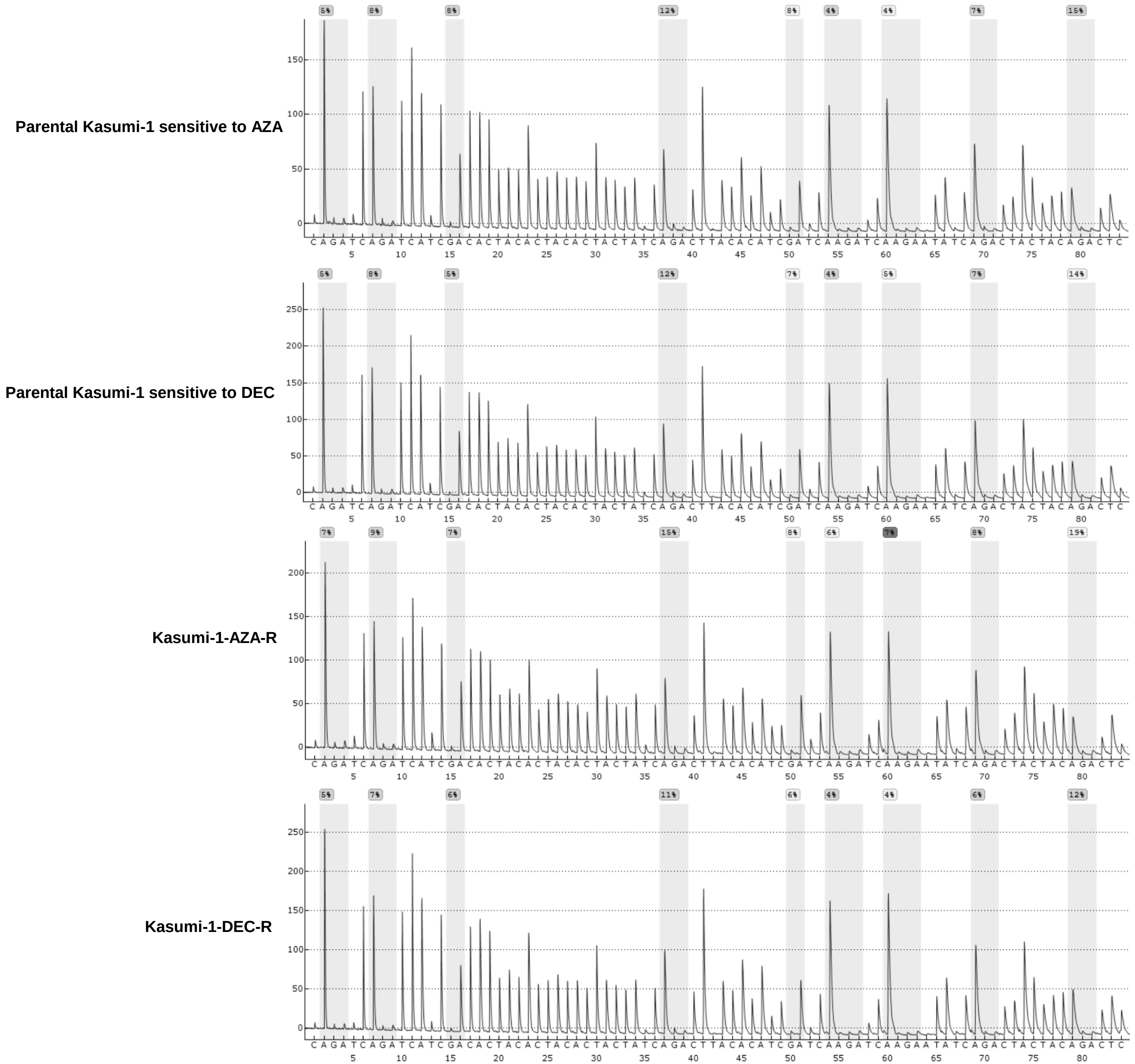

Figure S6. Pyrosequencing of *PTEN* in Kasumi-1 cell lines. Pyrograms of hypermethylated CpG upstream to *PTEN* in Kasumi-1-AZA-S, Kasumi-1-AZA-R, Kasumi-1-DEC-S and Kasumi-1-DEC-R. The fourth CpG analyzed by pyrosequencing was the same loci (cg10041390) detected to be hypermethylated in 850k whole methylome. AZA: Azacitidine; DEC: decitabine; Kasumi-1-AZA-R: Azacitidine-resistant Kasumi-1 cell line; Kasumi-1-DEC-R: decitabine-resistant Kasumi-1 cell line. The sensitive Kasumi-1 cells were treated separately with AZA and DEC at 1 μM for 48 hours followed by immediate harvest for the assessment
